# Oxidative phenotype induced by aerobic physical training prevents the obesity-linked insulin resistance without changes in gastrocnemius muscle ACE2-Angiotensin(1-7)-Mas axis

**DOI:** 10.1186/s13098-021-00693-w

**Published:** 2021-07-06

**Authors:** Bruno Vecchiatto, Rafael C. da Silva, Talita S. Higa, Cynthia R. Muller, Anna Laura V. Américo, Vanessa C. Fortunato-Lima, Marília M. Ferreira, Luiz Felipe Martucci, Miriam H. Fonseca-Alaniz, Fabiana S. Evangelista

**Affiliations:** 1 School of Arts, Science and Humanities, University of Sao Paulo, Av. Arlindo Bettio, 1000, Ermelino Mattarazzo, São Paulo, SP CEP 03828-000 Brazil; 2 Department of Experimental Pathophysiology, Faculty of Medicine, University of São Paulo, São Paulo, Brazil; 3 Heart Institute (InCor), Faculty of Medicine, University of Sao Paulo, São Paulo, Brazil

**Keywords:** Insulin resistance, Muscle metabolism, AMPK pathway, Angiotensin 1-7, Exercise

## Abstract

**Background:**

We investigate the effect of aerobic physical training (APT) on muscle morphofunctional markers and Angiotensin Converting Enzyme 2/Angiotensin 1-7/Mas receptor (ACE2/Ang 1-7/Mas) axis in an obesity-linked insulin resistance (IR) animal model induced by cafeteria diet (CAF).

**Methods:**

Male C57BL/6J mice were assigned into groups CHOW-SED (chow diet, sedentary; n = 10), CHOW-TR (chow diet, trained; n = 10), CAF-SED (n = 10) and CAF-TR (n = 10). APT consisted in running sessions of 60 min at 60% of maximal speed, 5 days per week for 8 weeks.

**Results:**

Trained groups had lower body weight and adiposity compared with sedentary groups. CAF-TR improved the glucose and insulin tolerance tests compared with CAF-SED group (AUC = 28.896 ± 1589 vs. 35.200 ± 1076 mg dL^−1^ 120 min^−1^; kITT = 4.1 ± 0.27 vs. 2.5 ± 0.28% min^−1^, respectively). CHOW-TR and CAF-TR groups increased exercise tolerance, running intensity at which VO_2_ max was reached, the expression of p-AMPK, p-ACC and PGC1-α proteins compared with CHOW-SED and CAF-SED. Mithocondrial protein expression of Mfn1, Mfn2 and Drp1 did not change. Lipid deposition reduced in CAF-TR compared with CAF-SED group (3.71 vs. 5.53%/area), but fiber typing, glycogen content, ACE2 activity, Ang 1-7 concentration and Mas receptor expression did not change.

**Conclusions:**

The APT prevents obesity-linked IR by modifying the skeletal muscle phenotype to one more oxidative independent of changes in the muscle ACE2/Ang 1-7/Mas axis.

**Supplementary Information:**

The online version contains supplementary material available at 10.1186/s13098-021-00693-w.

## Background

The unbalance between use and storage of lipid within skeletal muscle likely plays a significant role in the development of insulin resistance (IR) and type 2 diabetes mellitus (T2DM). Evidence in the literature showed that IR and T2DM are associated with lipid accumulation in the skeletal muscle [1] and that weight loss improves IR concomitant with decreased muscle lipid contents [2]. The excess of muscle lipids can impair the activity of proteins involved in insulin signaling, thus inhibiting the insulin-stimulated glucose disposal in the skeletal muscle [1].

The accretion of lipid within and around muscle fibers would seem to arise because of an increase in free fatty acid (FFA) uptake and/or decrease in fatty acid oxidation. Increases in FFA uptake into skeletal muscle were observed in animals and individuals with T2DM [1]. Furthermore, reduction in skeletal muscle oxidative capacity due to lower activity of enzymes such as citrate synthase and β-hidroxiacil-CoA desidrogenase (β-HAD) [3], damages in mitochondrial biogenesis and morphology can also lead to altered lipid partitioning toward storage favoring the development of IR and T2DM [1].

A useful strategy to prevent IR and T2DM is aerobic physical training (APT). We previously demonstrated that APT prevented obesity-linked IR in mice fed a cafeteria diet by improving lipolysis, preventing an increase in enzymes responsible for fatty acid esterification and by activating enzymes that improve fat oxidation instead of fat storage in the visceral white adipose tissue [4]. We also showed in the same animal model that APT reduced insulin signaling proteins and increased lipolysis signaling proteins in the subcutaneous white adipose tissue, and that cafeteria diet precluded the APT-induced thermogenic response [5]. Both studies showed mechanisms by which adipose tissue collaborates for the prevention of obesity and IR, however the role of skeletal muscle in this response is not fully understood.

APT can modify the skeletal muscle phenotype to one more oxidative because it improves the rate of glucose transport into skeletal muscle, the fatty acid transport into the mitochondria [6], the expression of metabolic and mitochondrial genes [7], the activity of enzymes responsible to substrate oxidation, angiogenesis and fiber typing changes toward oxidative-twitch fibers [8]. The adaptability of skeletal muscle is also associated with mitochondrial plasticity determined by network remodeling and continuous fusion and fission [1]. The intracellular mechanisms underlying the skeletal muscle response to APT are not complete revealed, but current findings suggest that increase in the AMP-activated protein kinase (AMPK) is a key regulator of skeletal muscle oxidative phenotype [6].

Different hormones and peptides including the renin angiotensin system (RAS) have been investigated as a target for reducing obesity and T2DM [9]. The activation of RAS axis composed of angiotensin converting enzyme 2 (ACE2), angiotensin 1-7 (Ang 1-7) and the Mas receptor (ACE2/Ang 1-7/Mas) can decrease body weight, improve lipid profile and metabolic syndrome, increase glucose uptake and reduce oxidative stress [10]. Ang 1-7 reversed Ang II or fructose-induced IR through improving insulin signaling in skeletal muscle, adipose tissue, and liver from rats [11, 12]. In contrast, ACE2 deletion exacerbated high-calorie diet-induced IR [13], and Mas deficiency is associated with metabolic syndrome in mice [14].

Considering that the expression of ACE2/Ang 1-7/Mas axis occurs in the skeletal muscle [15], the role of anti-obesity and anti-diabetic of this axis, and the potencial of physical exercise to modulate the RAS (12,13,14), the present study aimed to investigate the effect of APT on muscle morphofunctional markers and ACE2/Ang 1-7/Mas axis in an obesity-linked IR animal model induced by cafeteria diet. Our hypotheses was that APT could prevent obesity-linked IR through improvement in muscle oxidative phenotype associated with ACE2/Ang 1-7/Mas axis upregulation.

## Methods

### Animals and experimental design

Eight-week-old male C57BL/6J mice were assigned to four groups: CHOW-SED (chow diet, sedentary; n = 10), CHOW-TR (chow diet, trained; n = 10), CAF-SED (cafeteria diet, sedentary; n = 10) and CAF-TR (cafeteria diet, trained; n = 10). The standard chow diet contained 4% of kilocalories from fat, 55% from carbohydrate and 22% from proteins (Nuvilab®, Paraná, Brazil). The cafeteria diet contained 18.8% of kilocalories from fat, 55% from carbohydrate and 14.8% from proteins, which was able to induce increases in adiposity and insulin resistance in mice [16]. Animals were maintained under the same housing conditions (12-h light/12-h dark cycle, temperature 22 ± 2 °C) with free access to tap water and food ad libitum. Diet and APT were introduced at the same time and maintained for 8 weeks. Forty-eight hours after the end of the last training session, the animals were killed with an intraperitoneal injection of thiopental sodium (4 mg/100 g body weight) following exanguination. The animal was weighed and then the skeletal muscles (gastrocnemius, soleus and plantaris) and fat pads (subcutaneous, periepididymal and retroperitoneal) were harvested and weighed. All in vitro assays were done with gastrocnemius muscle. The procedures were approved by the Ethics Committee of the School of Physical Education and Sport of University of Sao Paulo (protocol number 26/2009) and Ethics Committee in Research from School of Arts, Sciences and Humanities from USP (protocol number 001/2016).

### Aerobic physical training

CHOW-TR and CAF-TR animals were submitted to APT during the dark cycle (i.e., during their active period) on a motorized treadmill for 1 h/day at 60% of maximal velocity achieved in the exercise test, five times per week for 8 weeks. APT intensity was progressively increased and adjusted after the graded treadmill exercise test done in the fourth week. APT and diet were started simultaneously. To minimize the influence of the treadmill stress, sedentary mice were placed on the treadmill for 5 min twice weekly at 0.3 km/h during the experimental protocol.

### Exercise testing and indirect calorimetry

Exercise capacity was assessed before, in the fourth and eighth weeks of APT using a progressive test without inclination on a treadmill as described by Ferreira et al. (2007) [17]. Initial velocity of treadmill was 0.4 km/h, and every 3 min, the velocity was increased by 0.2 km/h until animal exhaustion, which was characterized by the moment when the animal keep continuous contact with the shock grid for 5 s. The contact was defined as the continuous contact of any part of the animal in the shock grid for 5 s [18]. In the 4th week trained groups accomplished the test for running velocity adjustment.

The same test protocol was used to measure the metabolism performance by indirect calorimetry using Oxylet System (Panlab, Barcelona, Spain). After the end of APT, the mice were acclimatized in the system and volumes of oxygen consumption (VO_2_) was measured during resting (VO_2_ rest). Then the treadmill test was performed, and volumes of VO_2_ and carbon dioxide production (VCO_2_) were continuously measured during exercise test until the animal reaches the exhaustion. The maximum VO_2_ (VO_2_ max) was considered the average of VO_2_ obtained in the last stage of the test. The non-protein respiratory exchange ratio (RQ), a measurement of metabolic substrate preference, was calculated as the molar ratio of VCO_2_ to VO_2_ in the last stage of the test. The running intensity at which VO_2_ max was reached (iVO2 max) was measured as described by Machado et al. [19] and the reserve oxygen uptake (VO_2_R) was determined by the difference between VO_2_ max and VO_2_ rest. The rate of oxidation of carbohydrate (CHO) and lipids (LIP) were calculated as described by Ferrannini [20] CHO = (4.55 × VO_2_) − (3.21 × VCO_2_) and LIP = (1.67 × VO_2_) − (1.67 × VCO_2_). Data are expressed as mg min^−1^ kg^−1^.

### Body weight and food intake

Body weight was measured weekly at the same time of day using a digital balance (Gehaka, Model BK4001, Brazil). Body weight gain was calculated as the difference between body weight measured at the beginning and at the end of the PT protocol. The 24-h food intake was determined weekly throughout the study in mice that were housed at four animals per cage.

### Glucose tolerance test (GTT) and insulin tolerance test (ITT)

GTT and ITT were performed after the experimental protocol. Both experiments were performed in awake animals at 08:00 a.m. and after a 8-h fast. The glucose load (2 g/kg body weight) was injected as a bolus intraperitoneally, and the blood glucose levels were determined in caudal blood sampled at 0, 15, 30, 60, 90 and 120 min after glucose infusion. The glucose concentration was determined using a glucometer (AccuChek Advantage Roche Diagnostics®). After 72 h of GTT test, a similar procedure was performed for ITT. The insulin load (0.75 U/kg body weight) was injected as a bolus intraperitoneally, and the blood glucose levels were determined in caudal blood samples collected at 0, 5, 10, 15, 20, 25 and 30 min after injection. The values obtained between 5 and 30 min were used to calculate the rate constant for the disappearance of plasma glucose (kITT) according to the method proposed by Bonora et al. [21].

### Western blot analysis

Samples of frozen gastrocnemius muscle were homogenized in an ice-cold lysis buffer containing 50 mM EGTA (pH 8.5), 50 mM EDTA (pH 8.0), 500 mM KCl, 500 mM MgCl2, 500 mM HEPES, inhibitors of phosphatases C2 and C3 (1:300, Sigma Aldrich), inhibitor of protease (1:300, Sigma Aldrich) and PMSF (1:1000, Sigma Aldrich). Samples were then incubated for 30 min at 37 °C and centrifuged for 30 min at 14.000 rpm at 4 °C. Protein concentrations of the homogenates were measured by the BCA method with a protein assay kit (PIERCE Biotechnology, Rockford, IL, USA) using bovine serum albumin as a standard. Aliquots of protein were subjected to SDS-PAGE. The membranes were incubated overnight at 4 °C with the following primary antibodies: p-AMPK (Thr172), AMPK, p-ACC (Ser79), ACC (Cell Signaling, Beverly, MA, with dilution 1:2000), PGC1-α, SIRT-1, Mfn1, Mfn2, Drp1 (Abcam, Cambridge, USA, with dilution 1:2000), and Mas (Santa Cruz, with dilution 1:250). Secondary antibody goat anti-rabbit was marked with HRP (Invitrogen, New York, USA) (1:3000 in solution of skimmed milk at 5%) and for normalization the antibodies β-actin and GAPDH (Abcam, Cambridge, USA) were used. The signal on the membrane was detected via the peroxidase reaction in the ECL solution using an Image Quant LAS 4000 mini system (GE Healthcare Life Sciences®). Band intensities were quantified based on optical densitometry measurements using the Image J program (version 1.43 for Windows).

### Fiber typing

Muscle gastrocnemius frozen in liquid nitrogen was serially sectioned (10 µm) in cryostat. Muscle sections were incubated for myofribrillar ATPase activity after alkali pre-incubation (myosin ATPase, pH 10.3) as previously described [22]. The myosin ATPase reaction was used to identify the muscle fibre type. Type I fibres reacted lightly and type II muscle fibres reacted deeply after pre-incubation at pH 10.3, respectively. Fiber type distribution were evaluated in gastrocnemius muscle (~ 300 and 500 fibres) in light microscopy at 20× magnification and analyzed by a computerized morphometric analysis system (Image Pro-Plus 4.1; Media Cybernetics, Silver Spring, MD, USA). The total number of each fiber type was counted to calculate the numerical fiber type composition (I, IIa and IIb). The results were presented as frequencies (%) of the types of fibers. All analyses were conducted by a single observer (RC da Silva) blinded to mice identities.

### Lipid and glycogen contents

Lipid content was measured using quantitative histochemistry of Oil Red O (Sigma-Aldrich) staining of gastrocnemius muscle according to Goodpaster et al. [23]. Tissue sections (thickness 5 µm) obtained in a cryostat were examined by light microscopy at 40× magnification and analyzed by a computerized morphometric analysis system (Image Pro-Plus 4.1; Media Cybernetics, Silver Spring, MD, USA). Lipid accumulation was counted in 5 images per animal and the results were presented as percentage of area occupied by lipid droplets. Skeletal muscle analyses were conducted by RC da Silva blinded to mice identities.

Muscle glycogen content was evaluated as described by Voltarelli et al. [24]. Approximately 50 mg of gastrocnemius muscle were digested in 30% KOH at 100 °C, followed by the addition of ethanol 100% at 100 °C for glycogen precipitation. Samples were cooled on ice and submitted to centrifugation (30 min at 3.500 rpm and 4 °C). The supernatant was then decanted off and the precipitated glycogen was obtained quantitatively by two successive extractions with trichloroacetic acid 5%. Glycogen was estimated colorimetrically with an anthrone reagent (0.2% solution in 95% sulphuric acid). Sample absorbance was read at 620 nm and compared with a standard glucose curve. The values are expressed in mg of glycogen per 100 mg of muscle.

### ACE2 enzyme assay

ACE2 activity was evaluated in gastrocnemius muscle using fluorescent peptides [25]. The samples were incubated with a solution of Abz-APK peptide (Dnp)-OH (Abz = ortho-aminobenzoic acid; Dnp = dinitrophenyl) (15 μM) in 0.2 M Tris–HCl buffer containing 200 mM NaCl (pH 7.5). The enzymatic activity was determined by measuring the fluorescence for 10 min (one reading per minute). Protein concentrations of the homogenates were measured by the BCA method with a protein assay kit (PIERCE Biotechnology, Rockford, IL, USA) using bovine serum albumin as a standard. The activity was expressed in uF/μg of tissue protein.

### Ang 1-7 peptide assay

The concentration of Ang 1-7 was evaluated in gastrocnemius muscle by ELISA using commercial kit (Biomatik Corp., Cambridge, Ontario) according to the manufacturer’s instructions. The result was expressed in pg/mL/μg of tissue protein.

### Statistical analyses

All values are expressed as mean ± SE. The results were compared among groups using two-way analyses of variance (ANOVA). The Tukey post hoc test was used to determine differences between means when a significant change was observed using ANOVA. A p value equal to or less than 0.05 was statistically significant (StatSoft®, Statistica v.10).

## Results

### APT prevented the obesity-linked IR

Table 1 shows the effects of diet and APT on body weight, weight of tissues and metabolic variables. No significant difference in body weight was found among groups in the beginning of protocol. However, final body weight was lower in CHOW-TR and CAF-TR groups compared with CHOW-SED and CAF-SED. CHOW-TR and CAF-TR (15.3% and 18.7%) mice also had lower body weight gain compared with CHOW-SED and CAF-SED (29.9% and 30.2%, respectively). CAF-SED group showed higher subcutaneous fat mass compared with other groups, and higher periepididymal and retroperitoneal visceral fat mass compared with CHOW-TR and CAF-TR. In addition, CHOW-TR group had lower periepididymal fat mass compared with CHOW-SED group. No significant difference in skeletal muscle weight was found among groups.

**Table 1:** Body weight, weight of tissues and metabolic variables of mice after experimental protocol

	CHOW-SED (n = 10)	CHOW-TR (n = 10)	CAF-SED (n = 10)	CAF-TR (n = 10)
Inicial body weight (g)	21.2 ± 0.5	21.1 ± 0.4	20.9 ± 0.3	20.9 ± 0.5
Final body weight (g)	27.3 ± 0.6	24.3 ± 0.6*	27.2 ± 0.6	24.7 ± 0.5*
Body weight gain (g)	6.3 ± 0.3	3.2 ± 0.5*	6.3 ± 0.6	3.8 ± 0.5*
Subcutaneous fat pad (mg/g)	11 ± 0.7	9.1 ± 0.8	15.4 ± 1.2^#^	10.3 ± 1.1
Periepdidymal fat pad (mg/g)	12.8 ± 0.7	9.6 ± 0.3^%^	15.4 ± 1.1^$^	11.1 ± 1.0
Retroperitoneal fat pad (mg/g)	2.9 ± 0.3	1.6 ± 0.1	4.2 ± 0.4^$^	2.9 ± 0.6
Gastrocnemius weight (mg/g)	11.1 ± 0.2	11.2 ± 0.09	10.6 ± 0.71	11.0 ± 0.18
Plantaris weight (mg/g)	1.20 ± 0.04	1.31 ± 0.07	1.15 ± 0.04	1.21 ± 0.04
Soleus weight (mg/g)	0.64 ± 0.03	0.71 ± 0.02	0.71 ± 0.07	0.71 ± 0.05
Fasted glucose (mg/dL)	121 ± 4.9	101 ± 5.1	133 ± 7.2^&^	124 ± 4.1
AUC (mg/dL/120 min)	28.333 ± 1133	25.975 ± 1305	35.200 ± 1076^#^	28.896 ± 1589
kITT (%/min)	3.4 ± 0.28	3.9 ± 0.33	2.5 ± 0.28^#^	4.1 ± 0.27
Food intake (g/animal/24 h)	3.68 ± 0.07	3.31 ± 0.07	4.09 ± 0.07	4.21 ± 0.10

Data are presented as mean ± SE

AUC: area under the curve; kITT: rate constant for glucose disappearance

p ≤ 0.05 *CHOW-TR and CAF-TR vs. CHOW-SED and CAF-SED; ^#^CAF-SED vs. CHOW-SED, CHOW-TR and CAF-TR; ^$^CAF-SED vs. CHOW-TR and CAF-TR; ^%^CHOW-TR vs. CHOW-SED; ^&^CAF-SED vs. CHOW-TR

Glucose tolerance and insulin resistance were evaluated at the end of the experimental period. Although CAF-SED group had higher fasting glycemia only compared with CHOW-TR group, the area under curve (AUC) was higher and kITT value was lower in CAF-SED group compared with other experimental groups, confirming that cafeteria diet induced glucose intolerance and insulin resistance and that APT prevented these responses. Food intake in grams assessed over a 24-h period did not differ among groups (Table 1).

### APT increased exercise tolerance

The metabolic parameters at running test can be seen in Table 2. There were no differences in VO_2_ rest, RQ, CHO and lipid oxidation. Trained groups showed an increase in time exercise performance (time until exhaustion) and IVO_2_ when compared with respective control groups, and CHOW-TR had higher values when compared with CAF-SED group. This represents that training was effective and animals were capable to perform the test for a longer time and reach higher intensity on VO_2_ max. Only CHOW-TR group presented higher values of VO_2_ max and VCO_2_ max when compared with sedentary groups, even with CAF-TR presenting an increase of 8.8% when compared to CAF-SED group. For the VO_2_R, both trained animals showed higher values when compared with CHOW-SED group, what allows longer time in aerobic metabolism and prolonged time to exhaustion.

**Table 2:** Metabolic response at running test after experimental protocol

	CHOW-SED (n = 10)	CHOW-TR (n = 9)	CAF-SED (n = 8)	CAF-TR (n = 8)
Exercise performance (minutes)	31.4 ± 6.1	38.2 ± 6.2*^#^	29.5 ± 2.8	36.8 ± 1.6*
VO2 rest (ml/min/kg)	48.6 ± 1.5	39.7 ± 2.4	49.5 ± 13.2	41.5 ± 4.1
VO2 max (ml/min/kg)	53.6 ± 6.0	60.2 ± 5.1*^#^	53.1 ± 5.4	57.7 ± 5.2
VCO2 max (ml/min/kg)	42.4 ± 5.1	48.0 ± 4.0*^#^	42.2 ± 2.7	45.7 ± 3.2
RQ	0.7 ± 0.0	0.8 ± 0.0	0.8 ± 0.0	0.7 ± 0.0
iVO2 (km/h)	1.8 ± 0.4	2.3 ± 0.4*^#^	1.7 ± 0.1	2.2 ± 0.1*
VO2R (ml/min/kg)	5.5 ± 2.2	20.5 ± 3.0^#^	11.5 ± 2.0	16.2 ± 2.9^#^
CHO oxidation (mg/min/kg)	107.7 ± 15.8	120.0 ± 19.5	106.2 ± 20.6	115.8 ± 16.5
Lipid oxidation (mg/min/kg)	18.6 ± 5.1	20.4 ± 7.4	18.2 ± 7.2	20.0 ± 5.3

Data are presented as mean ± SE

RQ: Respiratory exchange ratio; iVO2: running intensity at which VO2 max was reached; VO2R: reserve oxygen uptake; CHO: carbohydrate

p ≤ 0.05 *vs. CAF-SED, ^#^vs CHOW-SED

### Muscle oxidative phenotype is improved by APT

**Fig. 1:**
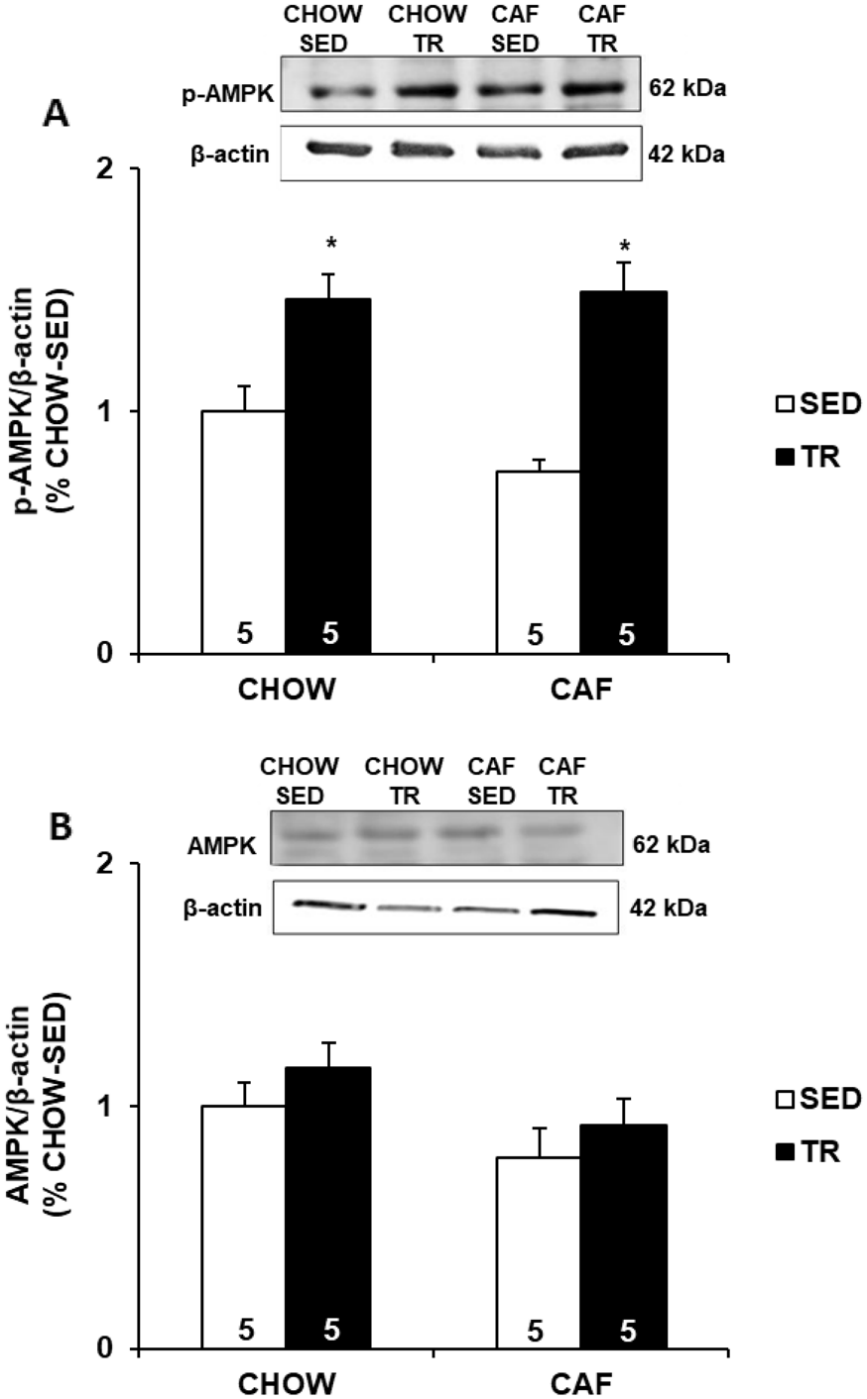
Expression of total AMPK (**a**) and p-AMPK Thr172 (**b**) in gastrocnemius muscle. Data are presented as mean ± SE. Immunobloting data are shown as the percentage of CHOW-SED (set to 1). p ≤ 0.05 *CHOW-TR and CAF-TR vs. CHOW-SED and CAF-SED. The results were compared by the two-way ANOVA

As showed in Fig. 1, it was not observed difference in the expression of total AMPK (Fig. 1a) among groups. However, both CHOW-TR and CAF-TR groups increased the expression of p-AMPK (Thr172) compared with CHOW-SED and CAF-SED groups (Fig. 1b).

**Fig. 2:**
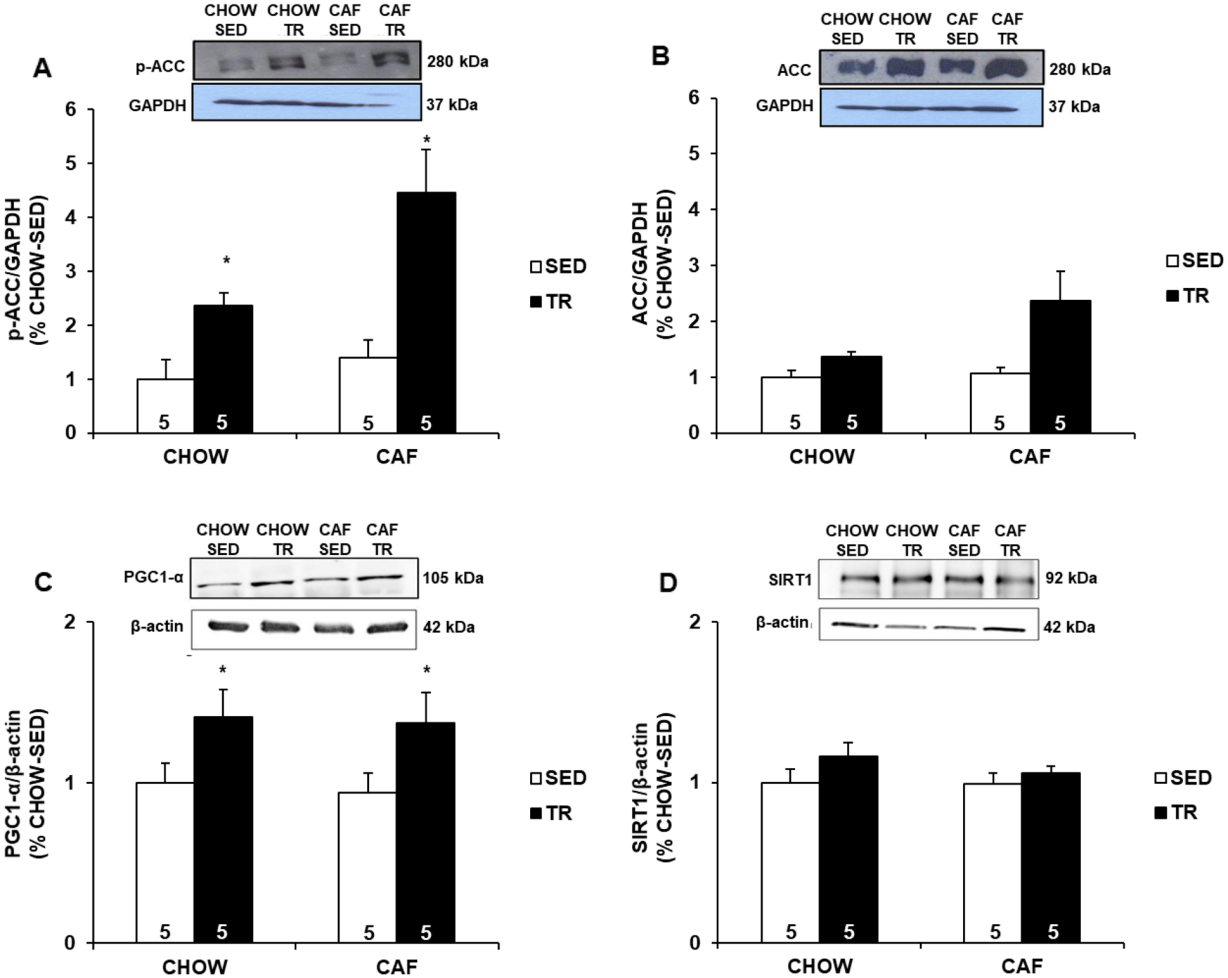
Expression of total ACC (**a**), p-ACC Ser79 (**b**), PGC1-α (**c**), SIRT1 (**d**) in gastrocnemius muscle. Data are presented as mean ± SE. Immunobloting data are shown as the percentage of CHOW-SED (set to 1). p ≤ 0.05 *CHOW-TR and CAF-TR vs. CHOW-SED and CAF-SED. The results were compared by the two-way ANOVA

It is known that ACC enzyme is a downstream target of AMPK, and as shown in Fig. 2a and b, CHOW-TR and CAF-TR groups had a higher expression of total ACC and p-ACC (Ser79) compared with CHOW-SED and CAF-SED groups. AMPK can directly phosphorylate and activate PGC1-α in the skeletal muscle, which is considered a master transcriptional regulator of genes involved in oxidative metabolism. In the present study, APT increased the protein expression of PGC1-α in CHOW-TR and CAF-TR groups compared with CHOW-SED and CAF-SED groups (Fig. 2c). PGC1-α can also to be activate by NAD+-dependent protein deacetylase SIRT1, however APT did not affect the amount of SIRT1 protein in the gastrocnemius muscle (Fig. 2d).

**Fig. 3:**
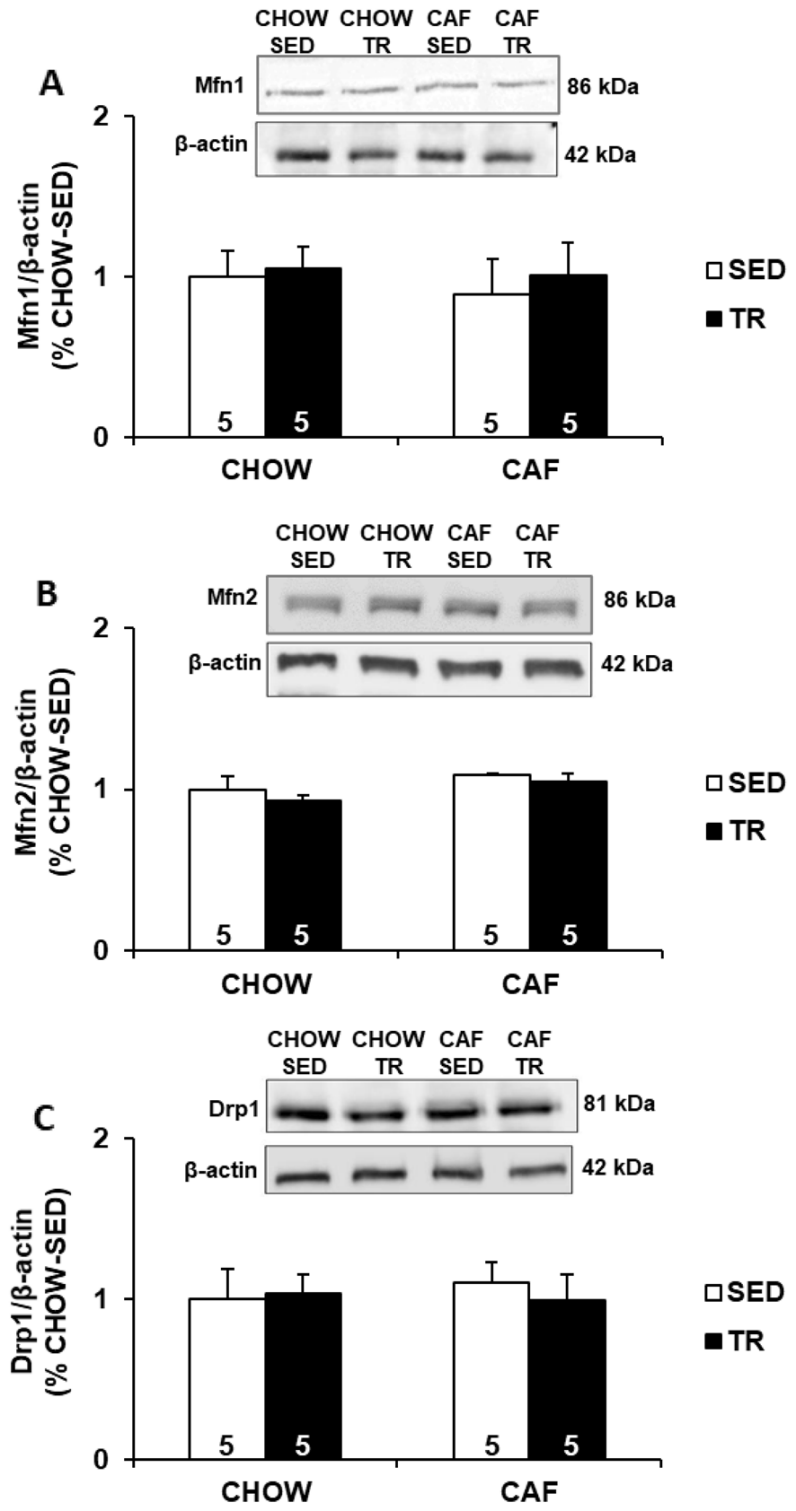
Expression of Mfn1 (**a**), Mfn2 (**b**), Drp1 (**c**) in gastrocnemius muscle. Data are presented as mean ± SE. Immunobloting data are shown as the percentage of CHOW-SED (set to 1). The results were compared by the two-way ANOVA

To verify whether the adaptability of skeletal muscle to APT would be associated with mitochondrial morphological plasticity, we evaluated the expression of proteins involved in mitochondria remodeling such as Mfn1 and Mfn2 (fusion) and Drp1 (fission). As showed Fig. 3a–c, APT caused no effect in the expression of Mfn1, Mfn2 and Drp1 in the gastrocnemius muscle.

**Fig. 4:**
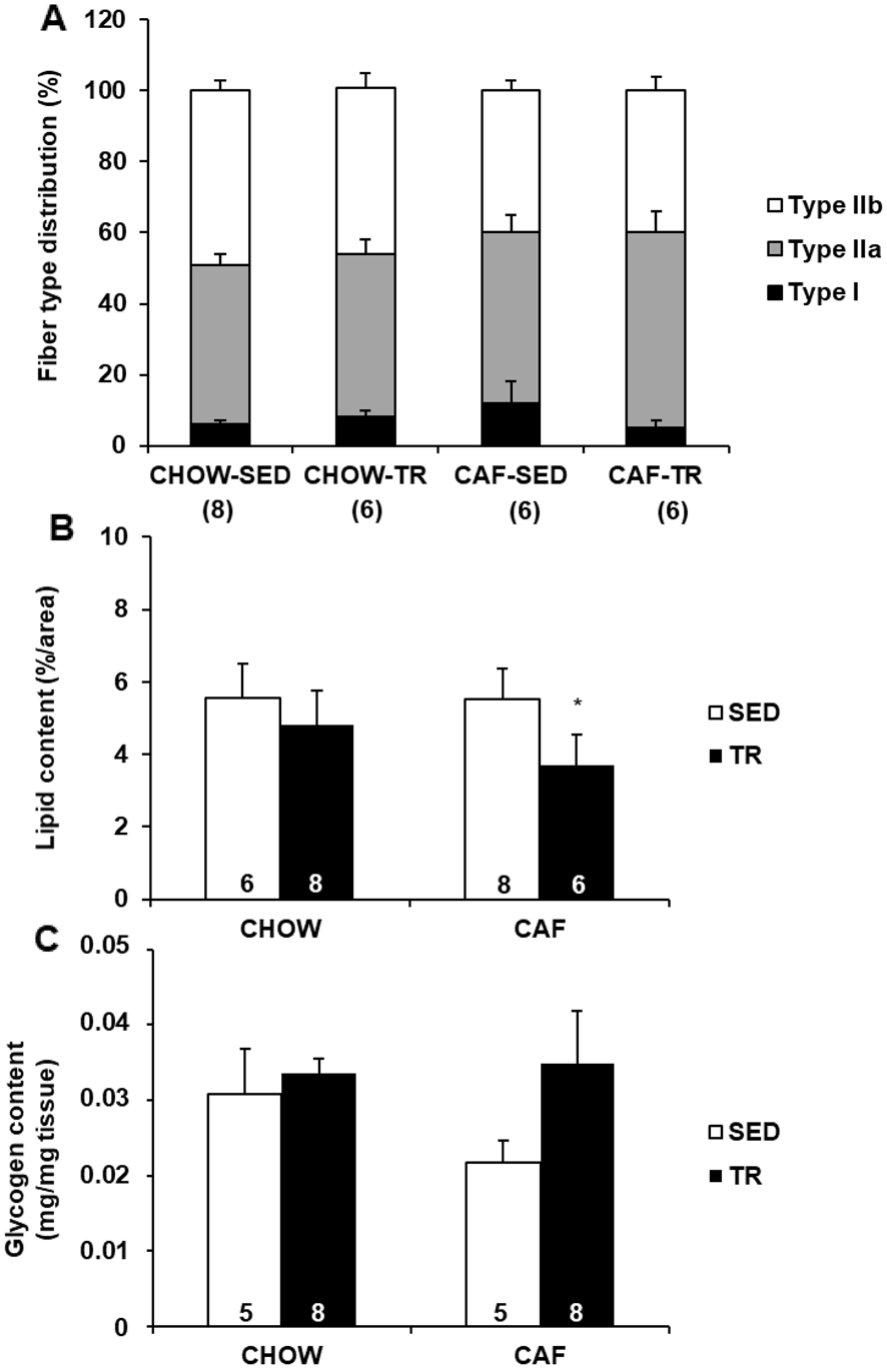
Gastrocnemius muscle fiber type distribution (**a**), lipid deposition (**b**) and glycogen content (**c**). Data are presented as mean ± SE. p ≤ 0.05 *CAF-TR vs. CAF-SED. The results were compared by the two-way ANOVA

To test whether preventive effect of APT against IR in CAF-TR group would be associated with increase in oxidative fiber types, we evaluated fiber type distribution in the gastrocnemius muscle. Myosin ATPase staining of muscle sections revealed that APT did not change fiber type distribution in both trained groups (Fig. 4a). No difference was also observed in CAF-SED group indicating that fiber type distribution was not modify by cafeteria diet (Fig. 4a). On the other hand, lipid content in gastrocnemius muscle was lower in CAF-TR group (3.71%/area) compared with CAF-SED group (5.53%/area), and no difference was observed in CHOW-SED (5.54%/area) and CHOW-TR (4.8%/area) groups (Fig. 4b). Glycogen content evaluated in the gastrocnemius muscle was not different among groups (Fig. 4c) (Additional file 1).

### Muscle ACE2/Ang 1-7/Mas axis did not change after APT

**Fig. 5:**
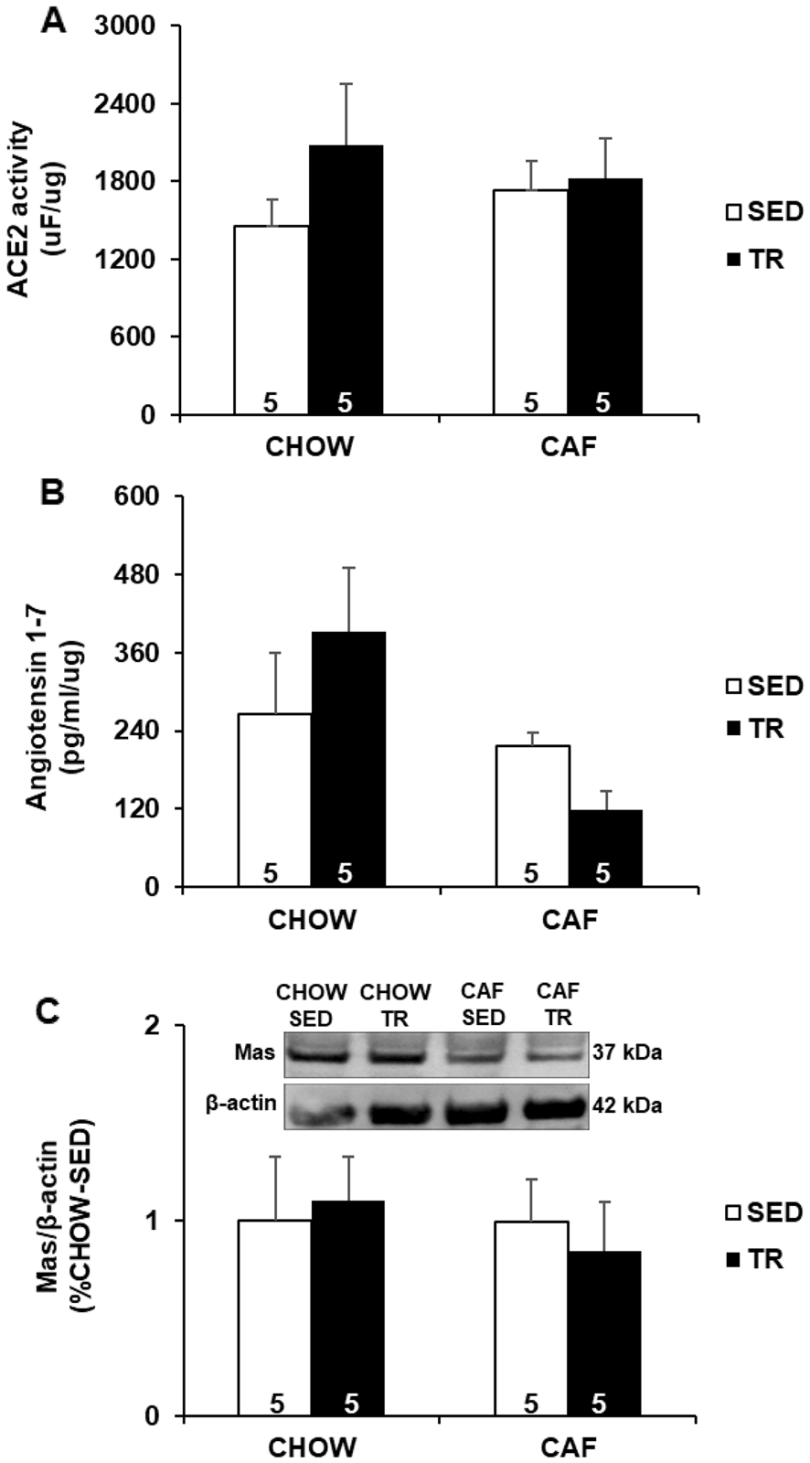
ACE2 activity (**a**), Ang 1-7 concentration (**b**) and Mas receptor expression (**c**) in gastrocnemius muscle. Data are presented as mean ± SE. Immunobloting data are shown as the percentage of CHOW-SED (set to 1). The results were compared by the two-way ANOVA

The components of RAS were measured in the gastrocnemius muscle and no statistical differences were observed in ACE2 activity (Fig. 5a), Ang 1-7 level (Fig. 5b) and Mas receptor protein expression (Fig. 5c). These results revealed that both APT and cafeteria diet did not change muscle ACE2/Ang 1-7/Mas axis.

## Discussion

In the present study, it was showed that APT increased the expression of oxidative proteins, decreased muscle lipid deposition and increased exercise test tolerance. These responses are indicative of improvement in the muscle oxidative phenotype and can be associated with the obesity-linked IR prevention by APT. However, the effects of APT were not associated with muscle ACE2/Ang 1-7/Mas axis changes.

The results found in body composition and glucose metabolism confirmed that cafeteria diet induces a significant body weight gain and adiposity, damages in glucose tolerance and insulin sensitivity in CAF-SED group. These responses were counteracted by APT in the CAF-TR group. The efficiency of APT in preventing obesity and IR has been demonstrated by our group [5, 16] and by other authors [26] and part of the mechanism responsible to improve the glucose metabolism is mediated by adipose tissue. In this context, APT can improve fat oxidation instead of fat storage, which reduces adiposity and improves the endocrine function of adipose tissue. Thus, there is a reduction in inflammatory adipokines secretion such as IL-6 and TNF-⍺ [27] and an increase in adiponectin secretion, which are related to better insulin sensitivity and glucose uptake by skeletal muscle [28].

Our study showed that APT did not change resting metabolic rate, which corroborates previous data published by our group [5] and others [29]. However, increases in resting energy expenditure after APT were observed in animals fed a high-fat diet [30], and can contribute to improve the energy balance and the body weight loss. The contradictory findings may be explained by differences in animal models, exercise, and diet protocols. Considering that food intake did not differ among groups, it is possible that higher daily energy expenditure due to APT session could be the main determinant of the lower body weight observed in CHOW-TR and CAF-TR groups.

Changes in the metabolic characteristics of skeletal muscle induced by APT reflect directly on the improvement of oxidative metabolism and consequently on the physical exercise tolerance. This effect has been indicated as a therapeutic goal for individuals and animals with metabolic diseases [26]. In our study, both trained groups showed better tolerance to physical exercise according to increased time until exhaustion, higher IVO_2_ and VO_2_R compared with sedentary groups, which indicates improvement in aerobic metabolism [19]. The CHOW-TR group showed higher VO_2_max when compared with both sedentary groups, but CAF-TR did not reach statistical difference, even with an improvement of 8.8% in this variable. One possible explanation for this result is the absence of treadmill inclination in the exercise protocol used during the tolerance test, since protocols with treadmill inclination are more sensitive to identify changes in endurance capacity then VO_2_max [18]. Furthermore, it is important to note that VO_2_max is based only on oxygen consumption, while IVO_2_ is determined by the intensity and the time that VO_2_max was reached, which can better represents the aerobic performance response [19].

One the major contribution to whole-body IR comes from the skeletal muscle, and the poor oxidative capacity is crucial to damage the insulin signaling. Thus, we investigated some effects of APT on skeletal muscle which may contribute to prevent IR. However, instead of studying a muscle with a large predominance of oxidative type 1 fibers such as soleus, that is typically used in physical training studies, we decided to investigate a muscle with more mixed fiber typing, such as the gastrocnemium muscle.

AMPK protein is a key regulator of energy metabolism because when activated can improve the carbohydrate metabolism and fatty acid oxidation [6]. In our study, CHOW-TR and CAF-TR groups presented higher levels of p-AMPK and the downstream target protein p-ACC. It is known that p-AMPK phosphorylate ACC enzyme and this decreases the production of malonyl-CoA, which controls the oxidation of fatty acids by inhibiting fatty acid transport via carnitine palmitoyltransferase 1. Although we did not measure fatty acid oxidation, the lower lipid deposition observed in the muscle of CAF-TR groups may indicate the improvement of oxidative metabolism.

AMPK also can directly phosphorylate and activate PGC1-α, which is considered a master transcriptional regulator of genes involved in oxidative metabolism. In fact, increases in the expression of PGC-1⍺ are associated with improvement in oxidative phosphorylation, mitochondrial biogenesis and generation of oxidative type 1 fibers [6]. We found that APT increased the protein expression of PGC1-α in CHOW-TR and CAF-TR groups, which confirms that APT can promote a more oxidative phenotype on skeletal muscle, favoring fatty acid oxidation [6]. In addition, PGC1-α can also to be activate by NAD+-dependent protein deacetylase SIRT1, however APT did not affect the amount of SIRT1 protein in the gastrocnemius muscle.

Increases in AMPK and PGC1-α can lead to improvement of mitochondrial dynamic. Thus, it was expected that APT could induce a higher fusion process, and lower fission process, resulting in a greater mitochondrial density and health maintenance [6]. Also, it was expected that cafeteria diet would change mitochondrial dynamics by promoting fission and blocking fusion process [31], with APT preventing these changes [7]. Due to unchanged expression of Mfn1, Mfn2 and Drp1 observed in our study, neither cafeteria diet or APT were capable to change mitochondrial fusion and fission revealing that in our experimental model, oxidative capacity was improved without changes in mitochondrial dynamics. Regarding to physical exercise, it is possible that the volume of exercise influences the mitochondrial dynamic response since changes in the volume of exercise from high to low reflects in loss of mitochondrial adaptations in skeletal muscle [32]. In addition, the effect of APT on the expression of fission and fusion proteins may be less robust in muscles with mixed fiber typing, since the effect of APT on mitochondrial dynamics was observed in soleus muscle [7].

The increase in p-AMPK and PGC1-α, and the decrease in p-ACC can mediate the improvement of oxidative metabolism and fatty acid oxidation. These changes resulted in lower lipid deposition, however were not associated with adaptations in the skeletal muscle fiber type and glycogen content. It is known that lipid accumulation in the skeletal muscle can damage insulin signiling and increase IR [1]. On the other hand, reduction in muscle lipid deposition as observed in the present study seems to be crucial to prevent obesity and IR, and can occur independent of changes in fiber typing.

The effect of APT on the RAS has been extensively investigated in the literature. Although different research indicated that the benefits induced by APT is associated with the upregulation of ACE2/Ang 1-7/Mas axis [26, 33], it is also known that APT downregulated the classical axis of the RAS [33] including angiotensin converting enzyme (ACE), angiotensin II (Ang II) and AT1 receptor (ACE/Ang II/AT1R axis), which is tipicaly hyperactivated in metabolic diseases such as obesity, DM and inflammation [34]. These data suggest that APT can induce a shift of the RAS balance towards the protective ACE2/Ang 1-7/Mas axis relative to ACE/Ang II/AT1R axis. In the present study, we investigated only the muscle ACE2/Ang 1-7/Mas axis and no significant changes were observed in both CHOW-TF and CAF-TF groups. Of course, we cannot exclude a possible effect of APT on the muscle ACE/Ang II/AT1R axis since it was not investigated here.

In a previous paper, rats fed a high-fat diet and trained for 12 weeks reduced body weight, improved glucose tolerance, IR, insulin signaling, and lipid profile. These responses were associated with lower AT1 expression and ACE/ACE2 ratio, higher Mas expression, and a shifted RAS balance toward the ACE2/Mas axis in gracilis muscle [33]. In another study, it was observed in an animal model of heart failure that APT normalized ACE2 and reduced ACE in plasma but did not change in the soleus and plantaris muscle [35]. The authors also found increased Ang 1-7/Ang II ratio in the plasma, and Ang 1-7 and Mas in both muscles [35]. Interesting that only a single bout of exercise increased Ang1-7/Ang II ratio in soleus muscle of healthy rats and improved the insulin sensitivity due to Ang 1-7 acting through Mas receptor [36]. These results revealed that differences in the muscles type, diet, exercise training protocols, and experimental animal model can explain the distinct results between our study and others. In addition, the APT induces unique responses in each skeletal muscle, and identifying the muscle-specific adaptations is important to determine how APT contributes to the prevention of obesity and IR.

## Conclusion

In summary, the present results provide evidence that APT prevents obesity-linked IR by modifying the skeletal muscle phenotype to one more oxidative independent of changes in the muscle ACE2/Ang 1-7/Mas axis. These findings have important clinical implications for individuals with high susceptibility to develop metabolic diseases because showed that APT is an important tool to improve the skeletal muscle oxidative capacity, which is crucial to avoid lipid deposition and insulin signaling damage.

## Supplementary Information

**Additional file 1.** Representative photomicrographs of gastrocnemius muscle incubated for myofibrillar ATPase activity to determine fiber typing and with Oil red to measure lipid deposition.

## Data Availability

The datasets analysed in the current study are available from the corresponding author on reasonable request.

## Abbreviations

IR: Insulin resistance
T2DM: Type 2 diabetes mellitus
FFA: Free fatty acid
β-HAD: β-Hidroxiacil-CoA desidrogenase
APT: Aerobic physical training
AMPK: AMP-activated protein kinase
RAS: Renin angiotensin system
ACE2: Angiotensin converting enzyme 2
Ang1-7: Angiotensin 1-7
Mas: Mas receptor
VO_2_: Oxygen consumption
VO_2_ rest: Resting oxygen consumption
VO_2_ max: Maximum VO_2_
RQ: Respiratory exchange ratio
VO_2_R: Reserve oxygen uptake
CHO: Carbohydrate
LIP: Lipids
GTT: Glucose tolerance test
ITT: Insulin tolerance test
kITT: Constant for the disappearance of plasma glucose
ACC: Acetyl-CoA carboxylase
Mfn1: Mitofusin 1
Mfn2: Mitofusin 2
Drp1: Dynamic related protein 1
PGC1-⍺: Transcriptional co-activator PPAR-gamma co-activator-1 alpha
SIRT1: Sirtuin 1
ACE: Angiotensin converting enzime
Ang II: Angiotensin II
AT1R: Angiotensin receptor 1
