# Supplementary material for: Oxidative phenotype induced by aerobic physical training prevents the obesity-linked insulin resistance without changes in gastrocnemius muscle ACE2-Angiotensin(1-7)-Mas axis
Source: Diabetol Metab Syndr. 2021 Jul 6;13:74. doi: 10.1186/s13098-021-00693-w (PMC8262010; doi:10.1186/s13098-021-00693-w)
Supplement: Supplementary file 1 — Additional file 1. Representative photomicrographs of gastrocnemius muscle incubated for myofibrillar ATPase activity to determine fiber typing and with Oil red to measure lipid deposition. [file 13098_2021_693_MOESM1_ESM.docx]

**Additional file**

Figure S1. Representative photomicrographs of histological sections of gastrocnemius muscle incubated for myofribrillar ATPase activity to determine fiber typing at 20X magnification. Light fibers are type I, deep fibers are type IIa and very deep fibers are type IIb.

Figure S2. Representative photomicrographs of histological sections of gastrocnemius muscle incubated with Oil red to measure lipid deposition at 40X magnification. Lipids droplets are stained in red.
